# Evaluation of Green Coffee-Roasting Biogas with Modeling Valorization of Possible Solutions

**DOI:** 10.3390/ijerph17196947

**Published:** 2020-09-23

**Authors:** Giuseppe Campo, Alberto Cerutti, Marco Ravina, Deborah Panepinto, Vincenzo A. Riggio, Mariachiara Zanetti

**Affiliations:** Department of Environment, Land and Infrastructure Engineering, Politecnico di Torino, 10129 Torino, Italy; giuseppe.campo@polito.it (G.C.); alberto.cerutti@polito.it (A.C.); marco.ravina@polito.it (M.R.); vincenzo.riggio@polito.it (V.A.R.); mariachiara.zanetti@polito.it (M.Z.)

**Keywords:** anaerobic digestion, coffee, biomass, byproducts, circular economy, climate change, environmental impact

## Abstract

According to the European Union Directive 2009/28/EC, the goals of obtaining 20% of all energy requirements from renewable sources and a 20% reduction in primary energy use must be fulfilled by 2020. In this work, an evaluation was performed, from the environmental and energy point of view, of anaerobic digestion as a valid solution for the treatment of the byproducts obtained from the coffee-roasting process. In particular, thermophilic anaerobic digestion tests were carried out. Output values from the laboratory were used as input for the MCBioCH_4_ model to evaluate the produced flow of biogas and biomethane and two different biogas valorization alternatives, namely, the traditional exploitation of biogas for heat/energy production and biomethane conversion. The results of the preliminary simulation showed that a full-scale implementation of the coffee waste biogas production process is technically feasible and environmentally sustainable. Furthermore, the performed analysis validates a general methodology for energy production compatibility planning.

## 1. Introduction

In recent years, there has been an increasing trend toward the efficient utilization of byproducts [1,2]. In the last few years, the possibility of saving energy by using resources that are considered waste has become more and more attractive to commercial plant management. The concept of waste has just begun to change. Agroindustrial products are included in this evolving scenario. One of the major agroindustrial products in terms of consumed amounts is coffee. Coffee is the second most traded product on the world market after fuel. The coffee market generates enormous volumes of waste, causing considerable economic and environmental concerns [3].

The consumption of coffee as a drink has been a custom for over 1000 years. The product began to be enjoyed in Europe in 1615, brought by travelers. Europeans began to exploit colonial territories in order to develop large coffee plantations. Currently, the largest exporter of coffee beans in the world is Brazil, with 40 million bags of 60 kg produced per year, followed by Vietnam, Colombia, and Indonesia. Specifically, South America is responsible for 43% of the world’s coffee production, followed by Asia with 24%, Central America with 18%, and Africa with 16%. It is estimated that 400 billion cups of coffee are consumed every year. Despite the financial crisis at the beginning of this century, coffee consumption did not decrease. The only fact that can be observed after 2008 is the change in the chosen quality by consumers, which is lower than in previous years [3].

The word “coffee” more appropriately refers to a wide range of products: fresh fruit, green beans, roasted beans, beverages, and instant coffee [4]. The first treatment of the product takes place in exporting countries. The main residues of these processes are so-called coffee husks or coffee pulp; depending on the types of processing, the total amount of these two initial kinds of waste are equal to 53 ± 12% of the total weight of the fresh fruit. Next, the processing continues in the industries of the purchasing countries, where coffee silver skin is another waste obtained from this kind of process, and it is equal to about 1 ± 2%. The remaining 45 ± 10% of spent wastes come from brewing the coffee. One of the biggest buyers of this product is Italy, preceded by Germany and Belgium. Italy is also, in turn, one of the largest exporters of roasted coffee, counting more than 800 roasters throughout the country, which, in 2019, generated a sales revenue of about 3.9 billion euros [5].

There are three types of processing waste generated in coffee-roasting plants: Green coffee powder, pellets, and roasted coffee powder [5]. They are properly defined as lignocellulosic biomass due to the high dry content of vegetable origin. The exploitation and management of coffee byproducts have been applied to various areas and finds new applications. Table 1 contains a list of byproducts associated with one or more methods of utilization found in the literature. Applications such as those linked to the antioxidant and protein properties of waste in cosmetics and pharmaceuticals are not lacking. Particular attention should also be paid to the production of animal feed: the presence of caffeine may discourage its use; however, preparations containing coffee byproducts are considered positive if introduced as food supplements and not as a substitute for a normal animal diet. Recently, research has been developed concerning the use of coffee waste for the production of bioplastics. This process requires that the residues are treated with solvents or biocompatible polymers to obtain a malleable and versatile material that is completely biodegradable. The research is currently being carried out by the Italian Institute of Technology (IIT) [6]. There are also several reports on the production of polyhydroxyalkanoate (PHA) bioplastics from spent coffee grounds [7,8,9].

Of interest is also the extraction of oil, in particular, from coffee grounds coming from residues of drink production, to be used as fuel. The new British start-up Bio-Bean is developing the possibility of using this fuel as a biofuel for buses [10].

Table 1 shows that the most reliable and efficient technologies that have been considered for energy recovery from these types of materials are direct combustion and anaerobic digestion (AD) [11,12]. In fact, all kinds of residues of coffee production can be managed by these two main technologies. The analyzed matrices have a high lower heating value on the one hand and, on the other hand, they can also provide essential minerals for the growth of anaerobic bacteria [5].

This work will evaluate anaerobic digestion as a possible alternative for the valorization of byproducts obtained from the coffee-roasting process [16,17]. The developed activity is a continuation of a previous study carried out by the same authors [5]. It should be emphasized that although the possible uses of all coffee byproducts are mentioned in the literature, there are few experiments on the roasting byproducts.

Different byproducts have different characteristics that make them more or less compatible with undergoing an anaerobic digestion process. Moreover, their availability in terms of volume must be adequate to allow their sustainable exploitation at a full scale. The applicability of coffee byproducts must thus be supported by both an experimental and modeling investigation. This integrated approach represents the novelty of the present work, where experimental analyses are matched with the application of modeling techniques to evaluate the two aspects together (productivity and scalability).

In order to perform an evaluation of the proposed technical solutions concerning biogas plants, a computational model developed at Politecnico di Torino Department of Environment, Land, and Infrastructure Engineering, called MCBioCH4, was employed. This model is able to integrate laboratory tests and to support the choice of technical solutions that maximize the energy efficiency and environmental sustainability of the process.

## 2. Materials and Methods

### 2.1. Substrate Origin

In this work, six coffee-roasting byproducts were tested in order to evaluate their anaerobic biodegradability. Table 2 shows the production of the tested substrates (raw powder from green coffee, pellets, and powder from the roasted coffee) generated by two different plants. Both analyzed plants are managed by the same Italian coffee producer.

### 2.2. Substrate Characterization

Standard methods [18] were employed for determining total solids (TS) and volatile solids (VS). CHNS equipment was adopted for the elemental analysis concerning carbon, hydrogen, nitrogen, oxygen, and sulfur. Mahler’s bomb was used for determining the higher heating value (HHV) of the three matrixes according to the calorimetric method. The theoretical methane potential values were calculated, taking into account Equation (1) [19].

(1) $$B_{th}=VS_{in}\times \frac{COD_{in}}{VS_{in}}\times 0.350\;\left[\frac{Nm^{3}}{kg\;COD}\right]$$

Given the CHNS analysis of a generic compound (C_a_H_b_O_c_N_d_), the *COD_in_/VS_in_* ratio was calculated, as in Equation (2):

(2) $$\frac{COD_{in}}{SV_{in}}=\frac{8\times \left(4a+b-2c-3d\right)}{\left(12a+b+16c+14d\right)}$$

In Equation (2), $COD_{in}$ is the concentration of the fed substrate in terms of chemical oxygen demand, and 0.350 is the maximum theoretical conversion of chemical oxygen demand (COD) to methane at standard condition (0 °C, 10^5^ bar) [20].

### 2.3. Anaerobic Digestion Tests

Two successive phases of experimentation were carried out: The first involved the laboratory study of the three matrices individually; the second phase considered two different mixes of the three matrices. The fractions composing the mixes are linked to the annual quantities produced in Plant A and Plant B. In particular, the mix from plant A is composed of 6.2% green coffee powder, 26.3% roasted coffee powder, and 67.5% pellets. The Plant B mix is composed of 9.2% green coffee powder, 42.9% roasted coffee powder, and 47.9% pellets.

Thermophilic conditions (55 °C) and laboratory scale were adopted for the anaerobic digestion tests performed in duplicate. In any case, the laboratory tests ended when the daily methane production was less than 1% of the cumulative biogas production, as advised in the German Guidelines VDI 4630 [21]. The thermophilic condition was selected among mesophilic conditions as a light increase of biogas production was recorded, but an increase in kinetics was also observed, allowing the digestion of the organic matter in around 20 days instead of 28 (data not showed).

The test performed on the single substrates had a total duration of 18 days, while the second phase test had a duration of 35 days. The first phase of AD tests was performed in batch mode using 8–2.8 L lab-digesters placed in a thermostatic bath. Each digester had a working volume of 2.0 L. The anaerobic environment was prepared by filling head-space digesters with nitrogen gas. Each digester was manually mixed for 60 s once a day. Two of the 8 anaerobic digesters were blank tests used to evaluate the amount of methane generated by the inoculum. In blank tests, the volume of inoculum added to the reactors was the same as that used for the tests with substrates.

The second phase of AD tests was performed in fed-batch mode using 6–6.0 L lab-digesters placed in a thermostatic bath. Each digester had a working volume of 4.0 L. The anaerobic environment was prepared by filling head-space digesters with nitrogen gas. Each digester was manually mixed for 60 s once a day (60 rpm). Two of the 6 anaerobic digesters were blank tests used to evaluate the amount of methane generated by the inoculum.

The produced biogas in each test was collected in a gas bag (maximum volume 5 L), and the composition (CH_4_, CO_2_, O_2_, others) was recorded every working day, from Monday to Friday, for the whole duration of the test [22].

The inoculum was provided by the ACEA plant, located in the Turin area, where the thermophilic digestion process is applied to the organic fraction of municipal solid waste. A biogas analyzer (Biogas Check, Geotechnical Instruments Ltd., Coventry, UK) was employed to determine biogas composition in terms of CH_4_, CO_2_, and O_2_, flushing 500 mL of the collected biogas. After the biogas characterization, the residual volume of the biogas was measured using the residual gas for the displacement of water. The volume of methane was calculated by multiplying the percentage of methane for the biogas volume in normal conditions.

### 2.4. Model Analysis of Batch and Fed-Batch Assay Results

Batch and fed-batch AD tests are commonly employed to assess the methane production of a substrate. However, the results of those tests depend on several parameters: the activity and origin of the inoculum, temperature, digestion time, and the ratio between the inoculum substrate and the hydrolysis coefficient. In order to compare different substrates or digestion modes in terms of methane production, it is necessary to identify some parameters that are capable of fully describing the biological process [20]. Because of this, the methane production data obtained from the batch and fed-batch tests were used to assess biochemical methane potential (B_0_) and the first-order hydrolysis rate constant (k) of the studied matrix and related mixes.

B_0_ is the maximum amount of methane that a substrate can produce after an infinite time; *k* is a first-order kinetic constant that is able to model the disintegration process. The anaerobic digestion consists of four steps: hydrolysis, acidogenesis, acetogenesis, and methanogenesis. Hydrolysis is the first step, and it is the only one where the microorganisms are not directly involved. This process is merely a surface phenomenon in which the particulate and polymeric matters are degraded through the action of esoenzymes. After hydrolysis, the smaller molecules produced by the process can cross the cell barriers [23]. The disintegration–hydrolysis phase is generally the rate-limiting step during the AD of particulate/complex substrates [24]. The evolution of cumulative methane production during a batch AD test B(t) can be modeled according to a first-order reaction rate, as in Equation (3).

(3) $$B\left(t\right)=VS\times V\times B_{o}\times \left(1-e^{-kt}\right)$$

where the parameters not previously mentioned are the following: *VS* is the concentration of volatile solids, and *V* is the working volume of the anaerobic reactor. In addition, it is also possible to estimate the absolute biodegradation (Y), as defined in Equation (4).

(4) $$Y=\frac{B_{o}}{B_{th}}\;\left[\%\right]$$

Known Y, the cumulative methane production obtained during a fed-batch test, can be modeled according to the system of equations reported in Equation (5).

(5) $$\{\begin{array}{c} \frac{dYVS\left(t\right)}{dt}=\frac{M_{in}\left(t\right)}{V}-k\times Y\times VS\left(t\right)\\ B_{d}\left(t\right)=Y\times VS\left(t\right)\times k\times B_{o}\times V \end{array}$$

*M_in_* is the *i*-th mass of substrate in terms of volatile solids fed during the fed-batch tests. The first-order kinetic models (Equations (3) and (5)) were implemented in Simulink—MATLAB^®^ 2020a software (Mathworks Inc., 1 Apple Hill Drive, Natick, Middlesex, MA, USA) to search for *B_0_* and *k*.

The optimal set of B_0_ and *k* values was obtained by minimizing the objective function (J), that is, the residual sum of squares (RSS) between the measured data and the model-predicted data, as in Batstone and coauthors [25,26].

Moreover, the *B_0_* and *k* parameters obtained from batch tests carried out on the three coffee byproducts were validated. Those parameters were used to predict the cumulative methane production of the two mixes of the anaerobically tested matrices.

### 2.5. Computation Model for Evaluation of Biomethane Solutions

A preliminary assessment of the full-scale applicability of the recovery process was carried out by introducing the results obtained in the laboratory into a biogas/biomethane evaluation model developed by the authors.

The MCBioCH4 model (the acronym of the biomethane computational model) takes into account three aspects:

- to obtain data concerning the productivity of biogas plants with the related possible gas flow rates;
- to know the energy consumption of the plant and the related remaining energy flows, which is useful for economic valorization (electrical- and/or thermal-produced energy, biomethane for transport use, and/or gas distribution grid);
- to take into account the total environmental impact of the system.

The design of MCBioCH4 was specifically addressed to the preliminary assessment and comparison of different potential plant configurations and technological solutions based on the implementation of default datasets derived from extended bibliographic research. The computing code was entirely developed using MATLAB^®^ 2020a software (Mathworks Inc., 1 Apple Hill Drive, Natick, Middlesex, MA, USA). MCBioCH4 is composed of three different modules for the calculation of mass, energy, and greenhouse gas (GHG) balance, respectively. The code estimates the production of biogas and the relevant losses that may be expected in the system.

Energy conversion through biogas combustion and/or biomethane production is simulated by the model. If the biogas combustion option is selected, the model simulates combustion in commercial combined heat and power plants (CHPs). The amount of thermal energy recovery can be specified. If the biomethane option is selected, the user is allowed to select the upgrading technology, as well as customize the efficiency and parasitic consumption of the upgraded system.

The following technologies are taken into account: pressure swing absorption, pressurized water scrubbing, chemical absorption with amine, and membrane permeation. The model is also able to simulate other upgrading technologies such as cryogenic separation. The model is equipped with default parameters that, alternatively, may be customized by the user. The results of the model are

- Mass and energy balance of the system;
- The greenhouse gas balance of the system, referred to an equivalent traditional system with fossil fuels.

For more details, refer to Ravina et al. [27]. The MCBioCH4 model was recently applied to the evaluation of the anaerobic digestion of mixed agriculture–zootechnic waste matter [28] and wastewater sludge treatment [29,30].

For the simulation of the case under study, the experimental results of thermophilic anaerobic digestion tests were considered. Full-scale methane production for Plant A and Plant B was estimated by considering the substrate production reported in Table 2. Regarding methane energy conversion, simulations were repeated, considering both direct onsite biogas combustion and biomethane upgrading. For the first option, an electrical efficiency of the reciprocating biogas engines equal to 0.40 was assumed. The availability of 8000 h/y was assumed for the CHP unit. For the biomethane option, considering the Italian legislation on incentives for biomethane production [31], an injection into the national gas grid was considered as the final destination. A biogas upgrading technology with selective membranes (MBs) was simulated. The main input factors and values considered in the simulations are reported in Table 3. Due to the absence of direct measurements, the amount of methane lost, the energy autoconsumption, and the efficiency of the system were estimated by using the default parameters set by the model. For more information on these parameters, refer to [27]. Equivalent CO_2_ emission factors were taken by [32].

## 3. Results

### 3.1. Substrate Characterization

The CHNS-O, B_th_, HHV, TS, and VS analyses are reported in Table 4. The results showed high TS and VS concentration values in the analyzed substrates. Moreover, the green coffee powder HHV had a value of 50.7% higher than the other two tested byproducts.

### 3.2. Anaerobic Digestion Tests

**Figure 1:**
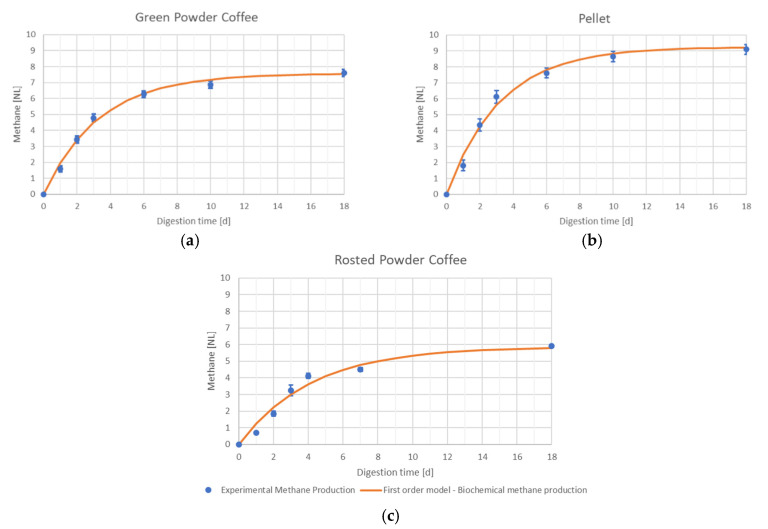
Anaerobic digestion (AD) batch tests. Measured points and simulated biochemical methane production curves. (**a**) green powder coffee; (**b**) pellet; (**c**) rosted powder coffee.

**Figure 2:**
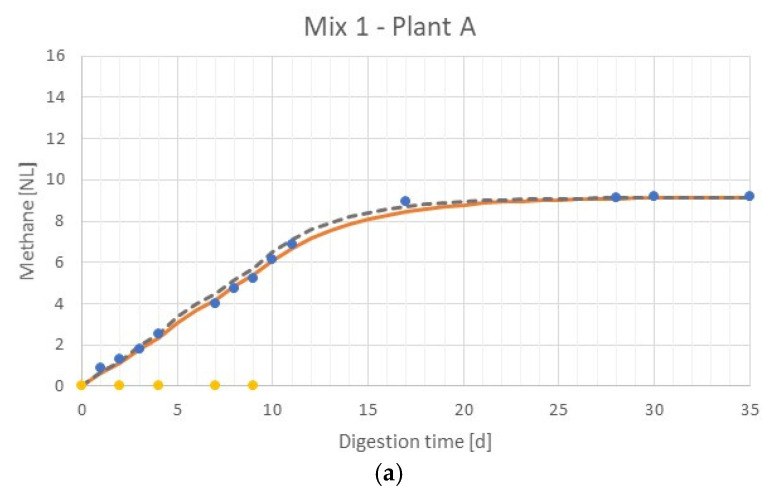

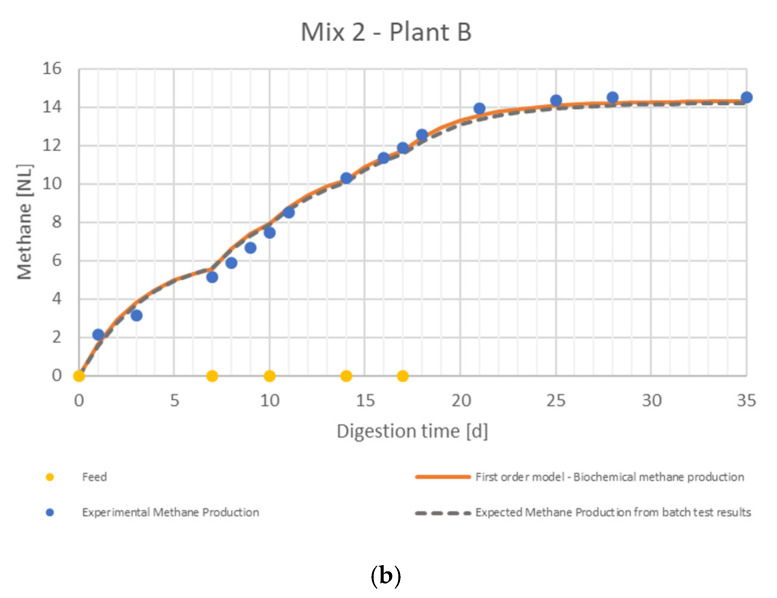
AD fed-batch tests. Measured points, simulated biochemical methane production curves, and expected biochemical methane production curves (validation of batch test results). (**a**) mix 1; (**b**) mix 2.

Figure 1 and Figure 2 show the experimental and simulated biochemical methane production profiles by the first-order kinetic model, which disclose the satisfactory fit of methane production data to this type of model. The results obtained in the first and second phases of anaerobic digestion tests, conducted in batch and fed-batch mode, are reported in Table 5. This table shows the biochemical methane potential (B_0_) and the absolute biodegradability (Y).

In Table 5, it is possible to notice that the biochemical methane production B_0_ was gathered from roasted coffee power, then there is the pellets’ specific methane production, and, at last, the lowest value is the one belonging to green coffee powder. In all the performed experiments, the methane concentration in biogas was about equal to 50% b.v.

The results of the AD tests carried out on the two mixes of byproducts are also reported in Table 5. The results show that the methane produced by the two mix tests are similar and linked to the SMPs of the three substrates digested in the first phase. Figure 2 displays the obtained cumulative methane productions during fed-batch AD tests. The same graphs show the expected cumulative methane productions found, considering the batch test results. These curves were calculated using Equation (5). In depth, B_0_ and k values were assumed from the weighted averages values of B_0_ and k, relative to each coffee byproduct tested, found during the batch tests; the results are reported in Table 5. Figure 2 displaces, in both the graphs, good accordance between the simulated biochemical methane production profiles by the first-order kinetic model and the expected one. Therefore, the results obtained in the batch tests are consistent and validated; in both fed-batch AD tests, they were able to predict the cumulative methane production trend.

According to the authors’ knowledge, there is no published research concerning the anaerobic digestion of coffee byproducts in thermophilic conditions, even though these kinds of residues are produced in high amounts in countries around the world. A recent study, Fiore et al. [11], has evaluated the specific biogas potential (SBP) of several types of food industry waste, also considering coffee byproducts. Anaerobic digestion was performed at the laboratory scale, with fed-batch mode and mesophilic conditions. Fiore et al. [8] measured the specific biogas production (SBP equal to 0.48 Nm^3^/kg VS, CH_4_/biogas = 0.55) of coffee byproducts (60% pellets, 40% roasted coffee powder), similar to the results presented in this work.

Baêta et al. [33] obtained a specific methane potential (SMP) for coffee husks equal to 0.063 Nm^3^/kg COD in mesophilic conditions by means of a BMP test; this result is lower than the results presented in this work (see Table 5; 0.14 Nm^3^/kg VS or 0.096 Nm^3^/kg COD).

Malovè et al. [31] reported a B_0_ for untreated pellets equal to NL/g VS, but this parameter reached a value three times higher by means of the adoption of a thermoalkali pretreatment with NaOH, heated for 3 h at 90 °C. The B_0_ of treated coffee pellets in Malovè et al. [31] was similar to this work’s results.

Lastly, Battista et al. [34] reported the specific methane production of a mixture made by pellets, roasted coffee powder, and green coffee powder (ratio 3:1:1), anaerobically digested (0.011 Nm^3^/kg VS). The result was much lower than the value obtained in this work, but the volatile solids reduction (24.92%) was similar to the experimental data.

### 3.3. Full-Scale Simulation Results

A preliminary analysis was performed in order to evaluate the feasibility of a full-scale anaerobic digestion plant for each industrial site. The results are shown in Table 6.

**Figure 3:**
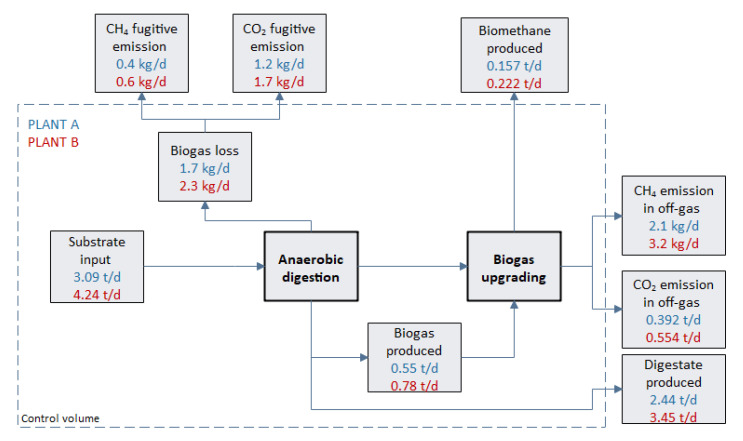
Mass balance of Plant A (in blue) and Plant B (in red).

**Figure 4:**
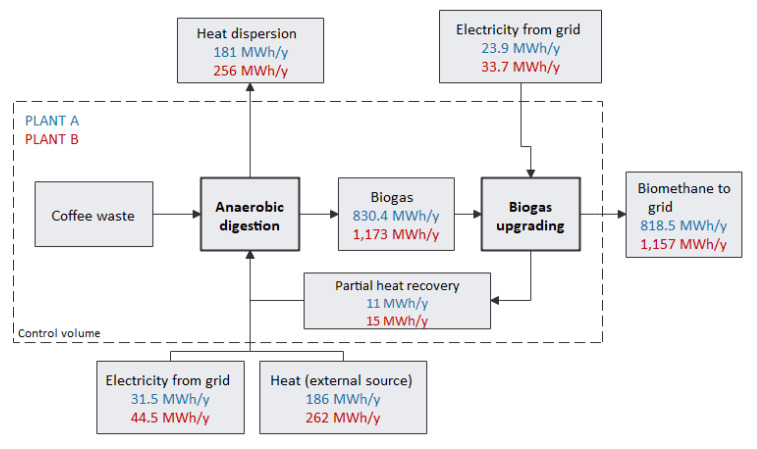
Energy balance of Plant A (in blue) and Plant B (in red).

The results reported in Table 6 were also implemented in the MCBioCH4 model to simulate biomethane production as an alternative solution to biogas direct combustion. The results obtained by running the mass, energy, and environmental modules for the two plants are reported in Figure 3 and Figure 4 and Table 7. The mass balance of Plant A and Plant B is reported in Figure 3. In this figure, the most relevant material flows are reported, which include both production and loss terms. Starting from an amount of input substrates (3.09 and 4.24 t/d for Plant A and Plant B, respectively), a low amount of biogas equal to 0.55 t/d and 0.78 t/d is produced. A limited amount of the produced biogas is lost in fugitive emissions from the system (1.7 and 2.3 kg/d for Plant A and Plant B, respectively). The net biogas flow is sent to the upgrading system, and biomethane is obtained. The expected biomethane production is 0.157 and 0.222 t/d for Plant A and Plant B, respectively. Due to the inefficiency of the separating system, a limited amount of the methane (2.1 and 3.2 kg/d for Plant A and Plant B, respectively) remains on the off-gas and is emitted into the atmosphere.

The mass balance of Plant A and Plant B is reported in Figure 4. In this figure, outgoing flows refer to the production terms and ingoing flows refer to the consumption terms. As reported in this figure, the AD system necessitates external sources of thermal energy and electricity. This indeed represents a limitation of the biomethane option. Thermal energy (186 and 262 MWh/y for Plant A and Plant B, respectively) is needed to maintain the digester’s temperature at a constant value, as well as to preheat the incoming substrates. This flow represents the highest consumption term. Electricity from the national grid is also needed by the auxiliary systems of the plant (31.5 and 44.5 MWh/y for Plant A and Plant B, respectively). The gross biogas energy content amounts to 830 and 1173 MWh/y for Plant A and Plant B, respectively. After the upgrading process, the net energy content of the produced biomethane is slightly lower than the gross amount, as part of the methane is lost in the off-gas. Additional consumption of electricity is also needed (23.9 and 33.7 MWh/y for Plant A and Plant B, respectively), while a small amount of heat is recovered from the biogas compression stage. Net energy production amounts to 818 and 1157 MWh/y for Plant A and Plant B, respectively.

The environmental balance reported in Table 7 shows that avoided emissions for the substitution of natural gas are higher than the emissions produced for process maintenance. An emission reduction of 81 t and 114.6 t CO_2eq_/y is estimated for Plant A and Plant B, respectively. This means that under the sustainability point of view, the production of biomethane would allow optimum exploitation of the energy contained in the waste matter. GHG emission for substrate handling and fugitive losses amount to 30.7 and 43.4 t CO_2eq_/y for Plant A and Plant B, respectively, and thus contribute significantly to the emission balance.

The results of the preliminary simulation show that, in principle, a full-scale implementation of the coffee waste biogas process is technically feasible and environmentally sustainable. This latter fact is generally recognized for the so-called third-generation biogas systems, i.e., systems that focus on waste valorization rather than biomass exploitation [35]. Considering the subsidies recently introduced by Italian regulations [36], it is expected that the presented configurations are also economically feasible solutions. Nevertheless, the economic balance of the proposed solutions will be evaluated in future studies.

## 4. Conclusions

In this work, thermophilic anaerobic digestion tests were carried out on two byproduct mixes from the green coffee roasting process. The fractions composing the mixes are linked to the annual quantities produced in Plant A and Plant B. The resulting B_0_ was 0.24 and 0.27 Nm^3^/kgVS from Mix A and Mix B, respectively. Starting from these results, the application of a full-scale anaerobic digestion plant was studied. Plant A could produce 150,000 Nm^3^ CH_4_/y, while Plant B could produce 226,000 Nm^3^/y. The power production of the two engines would be equal to 75 and 113 kWe, respectively, in Plant A and Plant B.

Output values from the laboratory were used as input for the MCBioCH_4_ model in order to evaluate an alternative valorization possibility for biogas, i.e., the production of biomethane. The results of the preliminary simulation showed that, in principle, a full-scale implementation of the coffee waste biogas process is technically feasible and environmentally sustainable. A net energy production of 818.5 and 1157 MWh/y was estimated for Plant A and Plant B, respectively. The produced biomethane was assumed to replace an equivalent amount of fossil natural gas, corresponding to an emission reduction of 81 and 114.6 t CO_2e_/y for Plant A and Plant B, respectively.

A preliminary evaluation of the possible solutions for energy recovery from green coffee roasting was performed in this study by means of an integrated environmental and modeling approach. This method may be applied to other similar cases.

## Figures and Tables

**Table 1:** Methods of use of coffee byproducts.

Utilization Methods	Coffee Husks, Pulp	Green Coffee Powder	Pellets	Roasted Coffee Powder	Coffee Grounds
Combustion	[4]	[12]	[12]	[9,12]	[12]
Anaerobic digestion	[12]	[12]	[12]	[9,12]	[12]
Substrate for the growth of edible mushrooms	[4]	-	-	-	[3]
Feed for livestock as a supplement for the diet of pigs, cattle, fish, sheep, and poultry	[4]	-	-	-	-
Fertilizers	[4]	-	-	-	[12]
Preparation of activated carbon	[4]	-	-	-	-
Biodiesel	-	-	-	-	[3,13]
Pharmaceutical industries	-	-	[3]	-	-
Cosmetic industries	-	-	[14]	-	-
Bioethanol	-	-	-	-	[3]
Production of a steak drink	-	-	-	-	[3]
Insulation material for buildings	-	-	-	-	[12,15]
Bioplastic	-	-	-	-	[14]

**Table 2:** Annual (2016) production of byproducts from the roasting process of green coffee in two analyzed plants.

PLANTS	Pellets (ton)	Green Coffee Powder (ton)	Roasted Coffee Powder (ton)
Plant A	780	70	280
Plant B	766	155	627

**Table 3:** Input values and parameters implemented in the MCBioCH4 model.

Input Parameter/Value	Plant A	Plant B
CH4 loss from digestion and conversion processes (%)	1.6	
Energy autoconsumption for thermal process sustainment (MWh/y)	181	845
Electricity autoconsumption, biogas section (MWh/y)	30.2	114
Upgrading system efficiency (%)	98.6	
Electricity autoconsumption, upgrading system (MWh/y)	22.9	109.32
Emission factor for natural gas consumption/substitution (gCO_2e/_kWh)	206	206
Emission factor for electricity substitution (Italian grid) (gCO_2e_/kWh)	337	337

**Table 4:** Elemental characterizations of the three considered matrices.

Matrix	N (%)	C (%)	H (%)	S (%)	O (%)	B_th_ (Nm^3/^kgVS)	HHV (MJ/kg)	TS (%)	VS (%)
Pellets	3.2	50.6	6.5	0.1	39.6	0.50	13.9	93.9	86.1
Roasted coffee powder	3.1	54.2	6.9	0.0	35.9	0.55	12.8	97.4	92.8
Green coffee powder	2.6	50.3	6.4	0.0	40.8	0.49	19.9	90.7	84.4

**Table 5:** Results of batch anaerobic digestion tests performed on a single matrix and mixed matrices.

Matrix	B_0_ (Nm^3^/kgVS)	k (1/d)	Y (-)
Pellets	0.22 (±0.01)	0.31	0.44
Roasted coffee powder	0.33 (±0.01)	0.24	0.60
Green coffee powder	0.18 (±0.01)	0.30	0.37
Plants A (mixed matrix)	0.24 (±0.01)	0.26	0.45
Plants B (mixed matrix)	0.27 (±0.02)	0.28	0.50

**Table 6:** Preliminary technical analysis.

Plants	Methane (Nm^3^/y)	Electrical Energy (kWh/y)	Electrical Power of Biogas Engine (kW)
Mix from Plant A	150,000	600,000	75
Mix from Plant B	226,000	900,000	113

**Table 7:** Output of the biomethane simulation with the MCBioCH4 model.

Input Parameter/Value	Plant A	Plant B
Gross biogas energy content (MWh/y)	830.4	1173.6
Net useful energy in biomethane (MWh/y)	818.5	1157.0
Thermal energy autoconsumption covered by external source (%)	100	100
Electricity autoconsumption covered by external source (%)	100	100
GHG emission produced substrates handling and fugitive CH_4_ loss (t CO_2e_/y)	30.7	43.4
GHG emission produced—electricity autoconsumption (t CO_2e_/y)	18.7	26.4
GHG emission produced—natural gas for thermal autoconsumption (t CO_2e_/y)	38.2	54.0
Total GHG emissions produced	87.6	123.8
Total GHG emissions avoided for natural gas replacement (t CO_2e_/y)	−168.7	−238.4
GHG emission balance (t CO_2e_/y)	−81.0	−114.6

## Abbreviations

AD	anaerobic digestion
B_th_	theoretical biochemical methane potential
B_0_	biochemical methane potential
CHNS	carbon hydrogen nitrogen sulfur
CHP	combined heat and power
COD	chemical oxygen demand
CO_2eq_	equivalent carbon dioxide
CRY	cryogenic separation
HHV	higher heating value
GHG	greenhouse gases
k	first-order hydrolysis rate constant
MEA	chemical absorption with amine solutions
MB	membrane permeation
PSA	pressure swing absorption
PWS	pressurized water scrubbing
SBP_ex_	experimental biogas production
TS	total solids
VS	volatile solids
Y	absolute biodegradability
